# Use, Utility, and User Experience of Cloud-Based Medical Imaging in Pulmonary Nodule Care in China: Mixed Methods Study

**DOI:** 10.2196/86745

**Published:** 2026-03-30

**Authors:** Ziguo Chen, Junhan Wu, Yunying Chen, Weitao Zhuang, Jiaxuan Huang, Weifeng Zhong, Zijie Li, Shuhuan Xie, Chaofan Liu, Guojie Lu, Guibin Qiao

**Affiliations:** 1 Department of Thoracic Surgery, Zhujiang Hospital, Southern Medical University, No. 253 Industrial Avenue Middle, Haizhu District, Guangzhou, 510280, China, 86 13602749153; 2 The Fifth Affiliated Hospital, Sun Yat-sen University, Zhuhai, China; 3 State Key Laboratory of Respiratory Diseases, National Clinical Research Center for Respiratory Disease, National Center for Respiratory Medicine, Department of Pulmonary and Critical Care Medicine, Guangzhou Institute of Respiratory Health, The First Affiliated Hospital of Guangzhou Medical University, Guangzhou, China; 4 School of Medicine, South China University of Technology, Guangzhou, China; 5 Shantou University Medical College, Shantou University, Shantou, China

**Keywords:** cloud-based medical imaging, pulmonary nodules, health care utilization, telemedicine, qualitative interview

## Abstract

**Background:**

The detection of pulmonary nodules (PNs) has increased with the use of low-dose computed tomography screening. Effective management requires timely longitudinal surveillance and reliable comparison with prior examinations, yet access to previous imaging across institutions is often fragmented, leading to delays and potentially unnecessary repeat scans and costs. Cloud-based medical imaging (CMI) solutions offer a potential means of improving access and facilitating cross-institutional data exchange. However, the adoption and utility of CMI in PN care, especially in China, remain underexplored.

**Objective:**

This study aims to evaluate the possession, use, and impact of CMI on health care utilization, patient knowledge, and financial burden, as well as to identify usability and interoperability barriers through qualitative investigation.

**Methods:**

A mixed methods cross-sectional study was conducted from October 2022 to May 2024. The study involved 701 patients with PNs who completed structured surveys, and 20 participants (10 patients and 10 physicians) were interviewed. CMI use was defined as self-reported ability to view radiological images on a mobile device. We compared CMI users and nonusers and estimated adjusted odds ratios using multivariable logistic regression, then applied 1:1 propensity score matching to examine associations between CMI use and health care utilization, costs, and patient perceptions, and qualitative interviews were analyzed for usability themes.

**Results:**

The study found that 611 (87.2%) out of 701 patients had obtained CMI, with 404 (57.6%) out of 701 patients actively using it. In multivariable analysis, older age was independently associated with lower CMI use (odds ratios 0.985, 95% CI 0.972‐0.999). After 1:1 propensity score matching, CMI users accessed more internet hospitals, consulted more physicians, and reported lower health care costs compared to nonusers. Users also demonstrated higher disease knowledge. Qualitative data identified key barriers, including poor system usability, limited retention time for images, and weak interoperability. CMI was perceived as beneficial for patient convenience and clinical efficiency, though concerns over image quality and system fragmentation were prevalent.

**Conclusions:**

While CMI is widely available, its usage remains suboptimal. Increased use is associated with enhanced health care engagement and reduced costs, suggesting that improving system usability and ensuring consistent access to imaging may help realize potential benefits of CMI. Future improvements should focus on ensuring long-term access, better retention protocols, and overcoming interoperability issues.

## Introduction

With the widespread adoption of low-dose computed tomography screening, the detection rate of pulmonary nodules (PNs) continues to increase [1]. Longitudinal imaging surveillance and comparison with prior examinations are foundational to PN management and are consistently emphasized in major guidelines [2,3]. In routine practice, patients often receive care across multiple institutions and seek additional opinions; when prior images are not retrievable promptly and reliably, repeat scanning and delays in care become likely. Evidence indicates that cross-regional or interinstitutional health information exchange (HIE) is associated with fewer duplicate imaging examinations and lower related costs [4,5].

In recent years, internet hospitals that commonly refer to hospital-affiliated online clinical services that extend routine care pathways through digital platforms, rather than stand-alone telemedicine encounters, have become an important complement to in-person clinics for remote consultation and follow-up. Real-world data suggest that their use is associated with fewer visits and lower costs [6,7], yet such encounters rely even more heavily on HIE. Enabling cross-institutional and cross-channel reuse of imaging requires cloud-based tools built on the Digital Imaging and Communications in Medicine (DICOM) or DICOMweb standards, which are collectively referred to as cloud-based medical imaging (CMI), and they support interoperable data exchange and network retrieval [8]. Recent implementations show that DICOMweb-based workflows can deliver web-based image viewing, improving visit efficiency. Recent surveys of web-based DICOM viewers suggest that DICOMweb support is increasingly common, but implementation depth and performance vary across products and settings [9]. Meanwhile, direct patient access to images is increasingly common. Although this can enhance knowledge and engagement, the absence of patient-facing explanations may trigger or amplify “scanxiety” [10]. Accordingly, information availability and interpretability represent distinct challenges. Recent studies showed that patient engagement with radiology reports and images is heterogeneous with respect to uptake, timing, and depth and differs across patients [11]. When patients review radiology results, they often encounter clinician-oriented terminology with limited general explanation, which may hinder comprehension and contribute to concern even when results are available in a timely manner [12,13]. A recent systematic review reported that patient-oriented reporting features, including plain-language summaries, glossaries, and visual aids, are associated with improved comprehension and satisfaction and may reduce avoidable anxiety [12].

In this imaging-dependent clinical context, timely retrieval and cross-channel reuse of imaging studies are prerequisites for effective longitudinal management. However, empirical evidence specific to populations with PNs remains sparse regarding CMI availability versus actual use, its associations with health care utilization and financial burden, user experience, and interoperability barriers. While digital image accessibility has been studied in general contexts, this research represents the first mixed methods study to focus on the unique PN care landscape in China. In this context, effective management is heavily dependent on long-term, high-frequency longitudinal imaging comparisons, making current limitations, such as brief data retention and system fragmentation, particularly impactful. Furthermore, unlike the unified patient portals common in other regions, the Chinese mobile health ecosystem is characterized by fragmented, QR code–based access, which presents distinct usability challenges. Beyond describing accessibility, this study provides a robust analysis of the correlations between actual CMI use and health care utilization, financial burden, and patient cognition, offering a holistic perspective on the “possession-to-use gap” that has not been previously characterized. This study aimed to characterize, within PN care, the landscape of CMI availability and use; quantify its associations with health care utilization and costs; assess relationships with disease perceptions; and, through qualitative inquiry, identify key usability and interoperability barriers to inform optimization and scale-up.

## Methods

### Study Design and Participants

We used an explanatory sequential mixed methods design. Survey findings informed the interview guide and purposive sampling, and integration was conducted through a prespecified comparison framework and meta-inferences based on convergence, complementarity, and discrepancies. Integrated findings are reported using narrative weaving that links quantitative results with qualitative themes and illustrative quotes. The reporting of the quantitative component follows the STROBE (Strengthening the Reporting of Observational Studies in Epidemiology) guideline, and the qualitative component was designed, conducted, and reported with reference to the COREQ (Consolidated Criteria for Reporting Qualitative Research). From October 2022 to May 2024, consecutive patients with PNs were recruited via a thoracic surgery e-consultation clinic on a national telemedicine platform [14]. In total, 701 participants completed the structured survey. Eligibility criteria were newly identified or undersurveillance PNs, age ≥18 years, and ability to complete the questionnaire. We excluded individuals who had already undergone resection or had a definitive pathological diagnosis.

To complement and contextualize the quantitative findings, we conducted semistructured interviews with a purposive, maximum-variation sample of 10 patients and 10 physicians. Patient participants varied by age (26‐73 y), sex, and educational level. Physician participants were thoracic surgeons spanning junior, mid-career, and senior ranks.

### Ethical Considerations

This study was approved by the Research Ethics Committee of Guangdong Provincial People’s Hospital (approval number: 2025-KY-122‐01). All participants provided written informed consent under institutional ethics approval. All questionnaire and interview data were deidentified before analysis, and access was restricted to the research team. The research team collected questionnaire responses and qualitative interview materials; we did not collect, download, store, or process participants’ DICOM files or other raw radiology image files as part of the study procedures. Participants received no compensation for participation.

### Measures and Variables

In China, CMI is commonly obtained or shared as DICOM files viewable in a browser, QR codes appended to radiology reports, or links on hospital websites. For QR code or web link access, the code or link typically directs patients to an institution-hosted or vendor-hosted web viewer. Depending on the implementation, the viewer may be DICOM-based (including zero-footprint viewers) or may rely on proprietary web formats; therefore, QR or link access should not be assumed to be DICOMweb-compliant. Some patients receive only a textual report and/or film, with no CMI access. We defined use of CMI as the self-reported ability to view radiological images on a mobile device. This measure does not differentiate between passive access and more active or intensive use, nor does it quantify frequency or depth of use. Sociodemographic and clinical variables included age, sex, education (primary, middle school, high school, junior college, undergraduate, and graduate), and surveillance status. Care-seeking and cost measures comprised the number of hospitals used (internet and offline), the total number of physicians consulted, and annual out-of-pocket expenditures (care-related fees, transportation, lodging). Self-efficacy and disease perceptions included perceived disease severity, level of disease awareness, and change in anxiety level.

### Qualitative Interviews: Design, Data Collection, and Management

We developed separate interview guides for patients and physicians (Multimedia Appendix 1). The guides covered CMI awareness and use, preferences of CMI format, perceived clinical benefits and limitations, workflow usability and pain points, psychological responses, and priorities for system improvement. Trained qualitative researchers conducted in-person interviews (20‐40 min). With permission, sessions were audio-recorded and accompanied by field notes. Recordings were transcribed verbatim, deidentified, and stored on password-protected devices. Texts were managed and queried in NVivo (version 11; QSR International Pty Ltd). Bilingual researchers translated quotations for manuscript presentation with dual review; discrepancies were resolved by revisiting the Chinese original. Additionally, interviews and analysis proceeded iteratively. During concurrent coding, we tracked the emergence of new codes and concluded interviewing when successive interviews no longer generated new major themes and the coding framework had stabilized.

### Data Analysis

#### Quantitative Analysis

Continuous variables were summarized using medians and interquartile ranges, whereas categorical variables were summarized as frequencies and percentages. The primary outcome was the use of CMI, defined as whether participants accessed and viewed their imaging data through CMI. Group comparisons were performed using chi-square or Mann-Whitney *U* tests. To quantify associations with CMI use, odds ratios with 95% CIs were estimated using univariable and multivariable logistic regression. To minimize confounding, propensity score matching was conducted with CMI use as the dependent variable. Prespecified covariates included age, sex, education, and surveillance duration. Nearest-neighbor 1:1 matching was applied using a caliper width of 0.02 times the SD of the logit of the propensity score. Covariate balance was assessed using standardized mean differences (<0.10). In the matched sample, health care utilization, financial burden, and disease perceptions were compared between CMI users and nonusers. Analyses were performed using SPSS Statistics (version 26.0; IBM Corp), and visualizations were created in GraphPad Prism (version 8.0).

#### Qualitative Analysis and Integration

Two researchers independently reviewed all transcripts, wrote reflexive memos, and conducted line-by-line open coding using in vivo codes where appropriate. After independent coding, the team discussed and resolved discrepancies; merged overlapping codes; refined code boundaries; and compiled a shared, version-controlled codebook. A constant comparative approach was used to group semantically related codes into categories within each participant group and to compare categories across groups using a patient-versus-physician matrix, identifying areas of convergence and divergence. Potentially disconfirming cases were examined to test the robustness of themes, which were iteratively refined based on clarity, coherence, and distinctiveness. Approximately 20% of the transcripts were double-coded for quality assurance, with disagreements resolved through discussion. An auditable analytic trail and reflexive notes were maintained throughout. Member checking was not undertaken. However, integration was performed through using the quantitative results to inform interview focus and an explicit joint display and narrative weaving to connect quantitative patterns with qualitative explanations and identify areas of convergence and discrepancy.

## Results

### CMI Possession and Use

#### Overview

Among 701 patients with PNs, 87.2% (n=611) had obtained CMI. Of these, 47.1% (n=330) acquired it by requesting from hospitals, while 40.1% (n=281) received it proactively from hospitals; 12.8% (n=90) had only reports or films without CMI. The main modalities were QR code or web link (n=352, 50.3%) and DICOM files (n=259, 36.9%). Overall, 57.6% (n=404) were CMI users, and 42.4% (n=297) were nonusers (Table 1).

**Table 1.** Possession and application of CMI^a^ among patients with PNs^b^.

Possession categories, application of CMI, and variables	Patients, n (%)
Possession of CMI	
No, I only got radiological report and/or film	90 (12.8)
Radiological reports and films	79 (11.3)
Radiological reports	11 (1.6)
Yes, I got it by applying to the hospital	330 (47.1)
DICOM^c^ files	259 (36.9)
Yes, hospitals took initiative to provide it	281 (40.1)
QR code or web links	352 (50.3)
Application of CMI	
Users of CMI	404 (57.6)
Nonusers of CMI	297 (42.4)

a CMI: cloud medical images.

b PN: pulmonary nodule.

c DICOM: Digital Imaging and Communications in Medicine.

#### Baseline Differences and Independent Correlates of CMI Use

In the unmatched sample (Table 2), users were younger than nonusers (median 46, IQR 38‐55 y vs median 48, IQR 39‐57 y; *P*=.001), and distributions differed for education (overall *P*=.004) and prior internet-clinic experience (overall *P*=.03). Sex (*P*=.60) and time since nodule detection (*P*=.09) did not differ. In multivariable logistic regression (Table 3), age was independently and inversely associated with CMI use (odds ratios 0.985, 95% CI 0.972‐0.999; *P*=.04), while education was not significant after adjustment (*P*=.21).

**Table 2.** Comparison of baseline characteristics between users and nonusers of CMI^a^.

Variable	Overall (N=701)	Users (n=404, 57.6%)	Nonusers (n=297, 42.4%)	*P* value
Age (y), median (IQR)	48 (39-57)	46 (38-55)	48 (39-57)	.001
Sex, n (%)				.60
Female	435 (62.1)	254 (62.9)	184 (60.9)	
Male	266 (37.9)	151 (37.1)	105 (39.1)	
Education attainment, n (%)				.004
Primary school	24 (3.4)	8 (2.0)	16 (5.4)	
Junior high school	68 (9.7)	41 (10.1)	27 (9.1)	
Senior high school	88 (12.6)	43 (10.6)	45 (15.2)	
Junior college	150 (21.4)	79 (19.6)	71 (23.9)	
Undergraduate	291 (41.5)	181 (44.8)	110 (37.0)	
Graduate	80 (11.4)	52 (12.9)	28 (9.4)	
Online consultation, n (%)				.03
Never	207 (29.5)	99 (24.5)	108 (36.4)	
Ever but need assistance	288 (41.1)	184 (45.5)	104 (35.0)	
Ever and not need assistance	266 (37.9)	121 (30.0)	85 (28.6)	
Time to the first detection of PNs^b^ (mo), median (IQR)	12 (3-32)	14 (4-32)	12 (2-33)	.09

a CMI: cloud medical images.

b PNs: pulmonary nodules.

**Table 3.** Binomial logistic regression analysis for influencing factors of CMI^a^ application in patients with PNs^b^.

Variable	Univariate analyses		Multivariate analysis	
	OR (95% CI)	*P* value	OR (95% CI)	*P* value
Age (y)	0.979 (0.967‐0.991)	.001	0.985 (0.972‐0.999)	.04
Time (mo)	0.998 (0.993‐1.004)	.58	—^c^	—
Sex (reference: female)		.60	—	—
Male	0.921 (0.677‐1.254)			
Education attainment (reference: primary school)	—	.02	—	.21
Junior high school	3.037 (1.142‐8.075)	.03	2.655 (0.989‐7.123)	.05
Senior high school	1.911 (0.742‐4.922)	.18	1.651 (0.634‐4.300)	.30
Junior college	2.225 (0.898‐5.513)	.08	1.833 (0.726‐4.626)	.20
Undergraduate	3.291 (1.363‐7.943)	.008	2.449 (0.973‐6.162)	.06
Graduate	3.714 (1.415‐9.750)	.008	2.583. (0.929‐7.178)	.07

a CMI: cloud-based medical images.

b PN: pulmonary nodule.

c Not applicable.

### Health Care Utilization, Costs, and Perceptions After Matching

**Figure 1.**
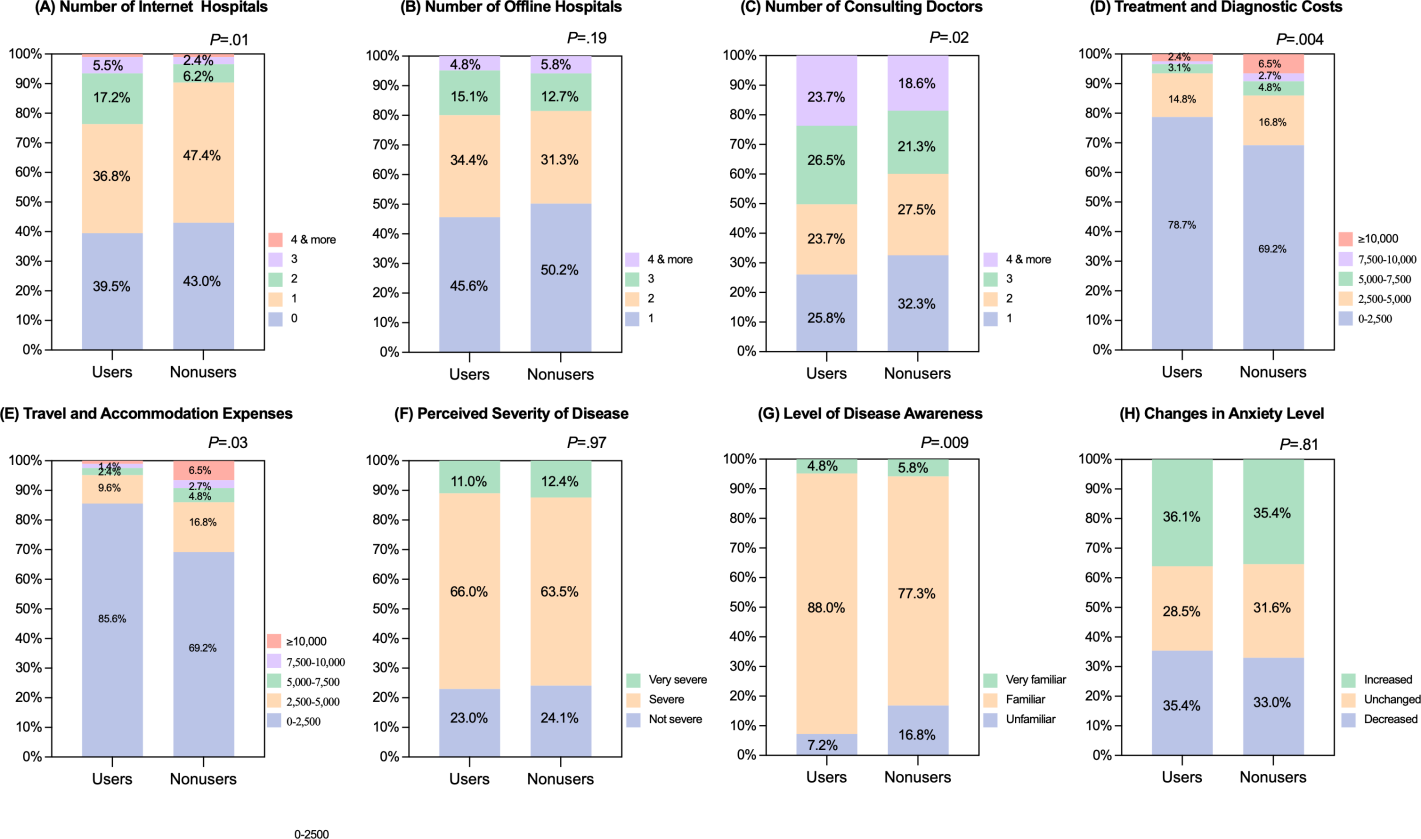
Health care utilization, costs, and perceptions after 1:1 propensity score matching. Note that 100% stacked bar charts compare cloud-based medical imaging (CMI) Users and Nonusers on 8 outcomes: (A) number of internet hospitals, (B) number of offline hospitals, (C) number of consulting physicians, (D) treatment and diagnostic costs, (E) travel and accommodation expenses, (F) perceived disease severity, (G) level of disease awareness, and (H) change in anxiety level. Bars show the percentage distribution of categories within each group (cost bands: Chinese Yuan [CNY] 0‐2500, 2500‐5000, 5000‐7500, and 7500‐10,000; ≥10,000; other category labels as shown in panel legends; all costs reported in CNY were converted to US dollars using an exchange rate of US $1=6.87 CNY). *P* values above panels compare distributions between the groups using 2-sided chi-square tests. Significant differences were observed for internet hospitals (*P*=.01), consulting physicians (*P*=.02), treatment or diagnostic costs (*P*=.004), travel or accommodation expenses (*P*=.03), and disease awareness (*P*=.009); no differences were found for offline hospitals (*P*=.19), perceived severity (*P*=.97), or change in anxiety (*P*=.81). Baseline balance after matching is shown in Table S1 in Multimedia Appendix 2.

After 1:1 propensity score matching, baseline balance was achieved, the main directional differences observed prematching generally persisted (Table S1 in Multimedia Appendix 2) and the covariate balance before and after propensity score matching persisted in Multimedia Appendix 3 . In the matched cohort, as shown in Figure 1A, the distribution for number of internet hospitals (median 1, IQR 0‐1 vs median 1, IQR 0‐1; *P*=.01) used differed significantly between users and nonusers, whereas, as shown in Figure 1B, the number of offline hospitals (median 2, IQR 1‐2 vs median 1, IQR 1‐2; *P*=.19) did not differ. The number of physicians consulted (median 3, IQR 1‐3 vs median 2, IQR 1‐3; *P*=.02) is also displayed in Figure 1C. CMI users were significantly more likely to spend 0‐2500 Chinese Yuan (CNY; US $1=6.87 CNY) on treatment and diagnostic costs (229/291, 78.7% vs 201/291, 69.1%; *P*=.004) and on travel and accommodation expenses (249/291, 85.6% vs 229/291, 78.7%; *P*=.03), suggesting they incurred lower expenses compared to nonusers. As presented in Figure 1F-H, regarding disease perceptions, users reported higher levels of disease awareness (overall *P*=.009), whereas perceived disease severity (overall *P*=.97) and anxiety change (overall *P*=.81) did not differ (Figure 1).

### Stratification by Access Modality (DICOM vs QR Code or Web Link) Among Users

**Figure 2.**
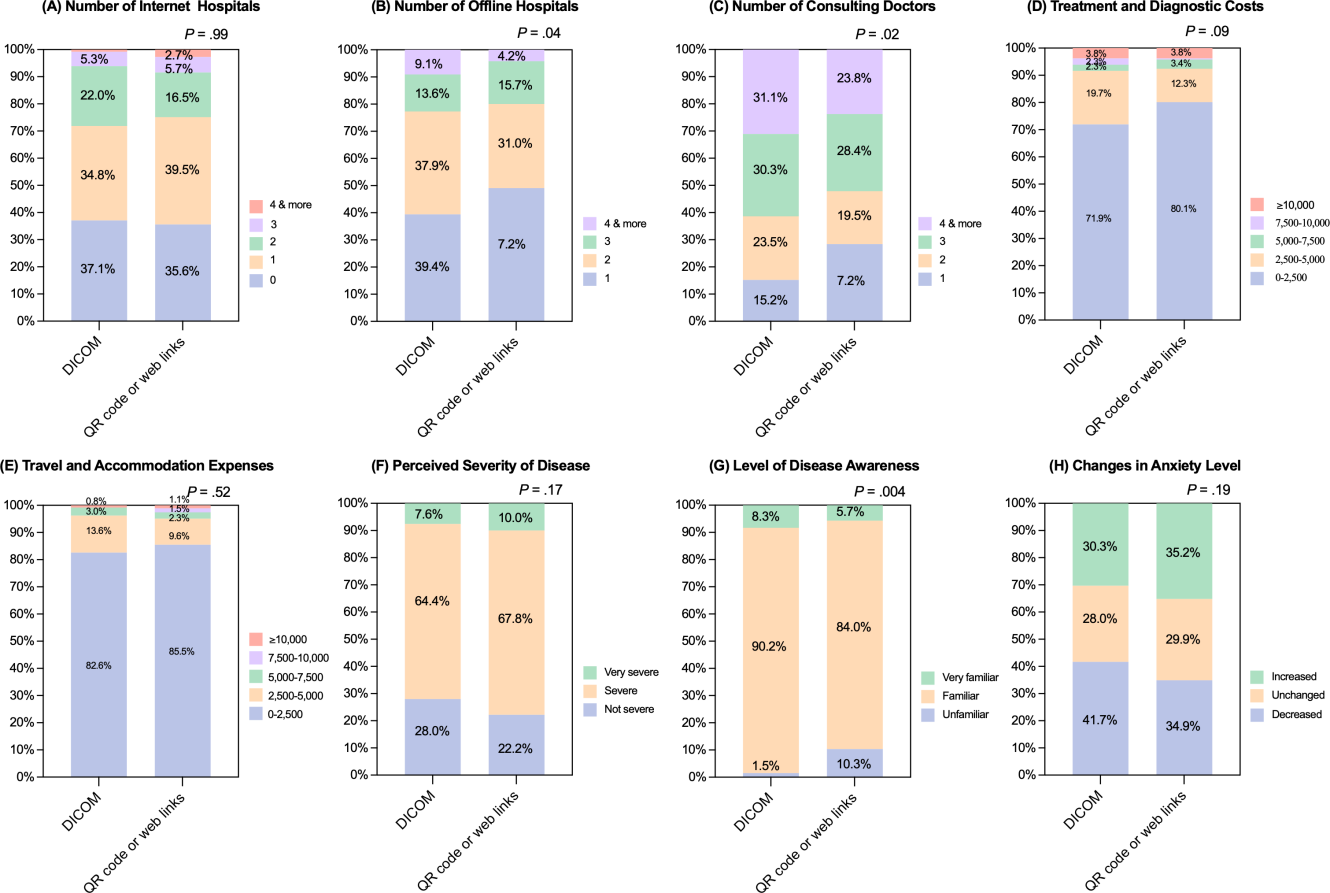
Outcomes among cloud-based medical imaging (CMI) users stratified by access modality (Digital Imaging and Communications in Medicine [DICOM] vs QR code or web link). Among CMI users only, 100% stacked bar charts compare those accessing the service via DICOM versus QR code or web link across the same 8 outcomes: (A) number of internet hospitals, (B) number of offline hospitals, (C) number of consulting physicians, (D) treatment and diagnostic costs, (E) travel and accommodation expenses, (F) perceived disease severity, (G) level of disease awareness, and (H) change in anxiety level. Bars display percentage distributions for each category (cost bands identical to Figure 1). *P* values above panels indicate 2-sided chi-square tests comparing modalities. Significant differences were seen for offline hospitals (*P*=.04), consulting physicians (*P*=.02), and disease awareness (*P*=.004). No significant differences were observed for internet hospitals (*P*=.99), treatment or diagnostic costs (*P*=.09), travel or accommodation expenses (*P*=.52), perceived severity (*P*=.17), or change in anxiety (*P*=.19).

Among CMI users, in Figure 2A, the DICOM group differed from the QR or link group in distributions of the number of offline hospitals (median 2, IQR 1‐2 vs median 2, IQR 1‐2; *P*=.04) and the number of physicians consulted (median 3, IQR 2‐4 vs median 3, IQR 1‐3; *P*=.02) as depicted in Figure 2B, whereas as shown in Figure 2C, the number of internet hospitals (median 1, IQR 0‐2 vs median 1, IQR 0‐2; *P*=.99) did not differ. The DICOM group had no difference in treatment and diagnostic costs (95/132, 72.0% vs 209/261, 80.1%; *P*=.09) and travel and accommodation expenses (108/132, 82.6% vs 223/261, 85.4%; *P*=.52) with the majority spending 0‐2500 CNY, but higher disease knowledge (overall *P*=.004) as presented in Figure 2G. The groups did not differ in perceived severity (overall *P*=.17) or anxiety change (overall *P*=.19; Figure 2).

### Integrated Findings Across Quantitative and Qualitative Strands

Across both strands, we observed a consistent possession-to-use gap: most participants obtained CMI, but only a subset reported using it. The interview data indicate that this gap reflects not only access but also day-to-day usability barriers and system limitations, including burdensome authentication steps, inconsistent workflows across hospitals, and short retention periods that make access links unreliable. After matching, the observed patterns in health care use and self-reported costs aligned with the convenience and efficiency benefits described in interviews, while psychological responses to viewing images varied in direction. A joint display linking key quantitative results with qualitative themes and illustrative quotes is provided in Table S2 in Multimedia Appendix 4.

### Qualitative Themes

#### Overview

We identified five overarching themes: (1) convenience and clinical value, (2) heterogeneous user experience and system usability, (3) limited retention time and poor interoperability, (4) psychological effects and communication, and (5) improvement priorities and governance. Representative quotes and themes are provided below (Table 4) and in Multimedia Appendix 1.

**Table 4.** Themes and representative quotes.

Theme	Representative quotes
Care convenience and clinical value	“I used to carry film to every visit; now showing the images on my phone is enough.” (Patient) [“When I follow up at another hospital, clinicians can view my prior images, saving many unnecessary steps.” (Patient) “Cloud-based imaging turns pulmonary nodule surveillance from discontinuous checkpoints into a continuous trajectory.” (Physician)
Divergent user experience and system usability	“Different hospitals require different accounts; I often can’t remember them.” (Patient) “When the connection is poor, the whole process fails.” (Patient) “There are too many steps to access the images; during busy clinics this disrupts workflow.” (Physician) “Window width and window level are difficult to adjust, which affects assessment of key details.” (Physician)
Limited retention and poor interoperability	“QR codes expire after a while, so at the next visit I have to start over.” (Patient) “Restrictions on the number of downloads are inconvenient.” (Patient) “Lack of interoperability creates information silos, making repeat imaging hard to avoid.” (Physician) “Without unified standards, retrieving outside images feels like assembling a jigsaw.” (Physician)
Psychological effects and communication	“Seeing the imaging changes makes me feel more in control.” (Patient) “There is too much medical terminology I don’t understand, which makes me more anxious.” (Patient) “We need to ‘translate’ images into conclusions and recommendations that patients can understand.” (Physician)
Improvement priorities and governance	“Ideally, a single platform would be usable nationwide.” (Patient) “Prioritize speed, clarity, stability, and privacy first; artificial intelligence (AI) features can come later.” (Physician) “It would be ideal to place history, reports, and images together in one ‘case folder.’” (Physician)

#### Theme 1: Convenience and Clinical Value

Patients and physicians agreed that CMI reduces patient burden and improves clinical efficiency. Patients emphasized not needing to carry films and smoother cross-institution visits; physicians highlighted longitudinal comparison of nodules, fewer unnecessary repeat scans, shorter visit times, and improved access for distant patients.

***Patient:*** I no longer carry a stack of films—showing the images on my phone is enough

***Patient:*** When I switch hospitals, prior images can be pulled up, and it’s easier for the doctor to decide on follow-up.

***Physician:*** Having previous images side-by-side makes surveillance decisions more coherent and reduces repeat CTs.

***Physician:*** For out-of-town patients, remote consults with shared images are much more efficient.

#### Theme 2: Heterogeneous User Experience and Usability

Experience diverged: younger or more digitally literate patients navigated smoothly, whereas older patients struggled with login, verification, account linking, scanning steps, bandwidth, and device limitations. Physicians cited slow loading, complex workflows, suboptimal image quality, windowing controls, and heterogeneity across hospital platforms.

***Patient:*** Some QR codes open directly; others require registration and verification—too many steps.

***Patient:*** If the network is poor, I just give up.

***Physician:*** When the clinic network slows, image loading disrupts the flow of consultation.

***Physician:*** Different platforms work differently; the learning burden prevents a stable routine.

#### Theme 3: Limited Retention and Interoperability Gaps

Short retention periods, expired links or QR codes, download limits, and weak cross-institution interoperability were common pain points. Patients could not retrieve older studies for long-term surveillance; physicians faced siloed platforms and resorted to repeat testing.

***Patient:*** Links stop working after a year or two, making longitudinal comparison difficult.

***Patient:*** Limits on how many times the link can be opened are especially inconvenient.

***Physician:*** Platform fragmentation leads to information silos and, inevitably, duplicate imaging.

***Physician:*** Without unified interfaces, retrieving outside images is inefficient.

#### Theme 4: Psychological Effects and Communication

CMI had bidirectional psychological effects: some patients felt reassured by ready access; others became anxious due to misinterpretation. Physicians noted that clear explanation is essential to avoid overinterpretation and undue worry.

***Patient:*** Seeing my images makes me feel more in control.

***Patient:*** The terminology is confusing; the more I look, the more anxious I feel.

***Physician:*** Without explanations, patients may overread images and worry unnecessarily.

#### Theme 5: Improvement Priorities and Governance

Patients and physicians broadly agreed on priorities but with different emphases. Patients wanted longer retention, removal of access limits, simpler flows, and cross-institution sharing. Physicians emphasized speed and clarity, unified standards or platforms, integration of history and reports, clear privacy and accountability, and gradual introduction of artificial intelligence–assisted localization and comparison.

***Patient:*** Ideally, images would be stored long-term without repeated registration.

***Physician:*** We need unified data standards and interfaces, integrating imaging, reports, and history into one workflow.

***Physician:*** Baseline requirements are clarity, loading performance, stability, and privacy; these should be solved first.

## Discussion

### Principal Findings

This mixed methods study shows that most hospitals can provide CMI in varied formats, yet a substantial possession-to-use gap persists. Compared with nonusers, CMI users engaged more with internet hospitals and physicians, reported lower care-related and travel or lodging costs, and self-reported higher disease knowledge. Qualitative findings pinpointed system usability, retention, timeliness, and interoperability as principal limitations. Overall, our results align with recent evidence on the accessibility and cost advantages of internet hospitals [6] and the association between interoperability and reduced duplicate imaging [5], offering practical directions for improving workflows.

Older age was independently associated with lower CMI use, whereas education was not significant after adjustment. This aligns with reviews showing that older adults face barriers with digital health tools—account/identity verification complexity, dense information, jargon, input burden, and screen design can all hinder adoption and sustained use [15,16]. Our interviews echoed concrete obstacles, such as multiplatform authentication, link expiry, and loading instability. For this population, the most effective improvements are those that reduce usability barriers rather than simply expanding access. Examples include single sign-on with unified entry points, clinic-side preloading combined with direct viewer access, caregiver proxy accounts, and concise instructional micro-guides, all of which have been shown to enhance eHealth participation among older adults [17-19].

Quantitatively, users interacted with more internet hospitals and physicians, while the number of offline hospitals did not change, suggesting that CMI may improve the efficiency and quality of care pathways rather than simply reducing in-person visits. Lower patient-reported costs for users are consistent with the reduced service frequency and improved accessibility observed with internet hospitals [6]. From a continuity-of-information perspective, integrated longitudinal viewers and HIE platforms reduce repeat imaging and patient burden [5], aligning with our cost-direction findings. Although users reported greater disease knowledge, perceived severity and anxiety did not differ, echoing prior work that immediate online access can increase knowledge but may sustain “scanxiety” in the absence of explanatory support [20]. Qualitative findings further contextualize these patterns by illustrating how CMI facilitates more efficient remote consultations and transforms surveillance into a continuous trajectory. These insights help interpret the observed cost reductions as a direct result of fewer unnecessary repeat scans and improved clinical efficiency during cross-institutional visits. The qualitative theme of limited retention and poor interoperability may also help explain the possession-to-use gap observed in our quantitative results, that even when patients have access to CMI, short retention periods and cross-institution barriers can reduce the practical usefulness of CMI and discourage actual use. Finally, the qualitative theme of bidirectional psychological effects explains why increased knowledge may not lower anxiety, as patients reported feeling more in control while simultaneously feeling confused by complex terminology in the absence of professional translation.

Stratification by access modality indicated advantages for DICOM over QR or link access in engagement and knowledge—plausibly due to DICOM’s interactivity, longer-term retention, and standardization, which facilitate time-series comparison and remote consultation. Technically, mature DICOMweb workflows support browser-based import and zero-footprint viewing suited to both lightweight patient access and professional clinical review [9]. At a systems level, better regional informatics and interoperability correlate with improved health service performance [21].

Our interviews highlighted short retention periods, inconsistent rules, and fragile links and QR codes as major obstacles, which undermine in-clinic retrieval. Consistent with research on patient portals, stable and renewable access points with clear audit trails and proactive expiry reminders are foundational for practical use, especially among older adults [22]. Clinically, immediate simultaneous access to prior images during the visit is critical. Rather than adding new entry points, default patient-centered retrieval organized by patient, chronological timeline, and imaging series, together with online comparison, should be standard capabilities. Workflows based on DICOMweb and zero footprint viewers already enable standardized retrieval and reliable online comparison without local installation [8], and access to prior images reliably reduces repeat testing and delays [23].

Our findings suggest that interoperability and durable access in CMI are not only technical issues but also governance issues that require consistent rules for minimum retention periods, renewable access, audit trails, and accountability across institutions [24]. China’s national health informatization agenda has emphasized standardization and interoperability maturity certification, which provides an actionable foundation to operationalize imaging-specific retention and exchange requirements at scale [25]. Building on recent analyses that identify standards, interoperability, legislation, and compliance as the core foundations of China’s digital health governance for the Healthy China 2030 initiative, the clinical management pathway for pulmonary nodule surveillance can be strengthened by aligning platform requirements with these governance elements [24,26]. Finally, equity-oriented implementation is needed to ensure that national digital health strategies translate into broadly accessible longitudinal imaging services rather than widening existing digital divides [26].

### Limitations

This cross-sectional design precludes causal inference. Visit counts, costs, and disease perceptions were self-reported and thus subject to recall and reporting bias. Recruitment through online and hospital settings may have overrepresented digitally literate individuals, limiting external validity. Consequently, the 57.6% usage rate likely represents an upper bound for this population, and the lack of regional stratification may mask even steeper barriers in underconnected or rural areas. Therefore, our findings are most applicable to digitally connected patients with PNs who engage in cross-institutional care and remote consultation and should be extrapolated cautiously to offline-only pathways and underserved settings. Although propensity score matching mitigated confounding, residual confounding cannot be excluded. The measure of CMI use does not distinguish passive access from more active use, and we did not capture frequency or depth of use. The qualitative sample was modest and did not include participant checking. Future studies could improve construct validity by incorporating intensity indicators ideally complemented by objective usage logs where feasible. Future multisite sampling, longitudinal, or quasi-experimental studies are also warranted to validate these findings and assess long-term adoption trajectories.

### Conclusions

In PN management, CMI is widely available yet remains underused. Actual use was associated with greater engagement in online care, lower related costs, and higher self-reported disease knowledge. Qualitative findings identified 3 actionable constraints—usability thresholds, unstable data retention, and inconsistent clinical viewing functions. Addressing these limitations requires ensuring renewable, auditable, and stable access, together with default support for longitudinal, in-browser comparison.

## Supplementary material

10.2196/86745 Multimedia Appendix 1 Interview guides.

10.2196/86745 Multimedia Appendix 2 Baseline characteristics for 701 patients in pulmonary nodules (PNs) after propensity score matching (PSM).

10.2196/86745 Multimedia Appendix 3 Covariate balance before and after propensity score matching.

10.2196/86745 Multimedia Appendix 4 Joint display of integrated quantitative and qualitative findings.

10.2196/86745 Checklist 1 COREQ checklist.

10.2196/86745 Checklist 2 STROBE checklist.

## Abbreviations

CMI: cloud-based medical imaging
CNY: Chinese Yuan
DICOM: Digital Imaging and Communications in Medicine
HIE: health information exchange
PN: pulmonary nodule
STROBE: Strengthening the Reporting of Observational Studies in Epidemiology

## References

[1] Gould MK, Tang T, Liu ILA (2015). Recent trends in the identification of incidental pulmonary nodules. Am J Respir Crit Care Med.

[2] MacMahon H, Naidich DP, Goo JM (2017). Guidelines for management of incidental pulmonary nodules detected on CT images: from the Fleischner Society 2017. Radiology.

[3] Wood DE, Kazerooni EA, Baum SL (2018). Lung cancer screening, version 3.2018, NCCN clinical practice guidelines in oncology. J Natl Compr Canc Netw.

[4] Jung HY, Vest JR, Unruh MA, Kern LM, Kaushal R, HITEC Investigators (2015). Use of health information exchange and repeat imaging costs. J Am Coll Radiol.

[5] Yuan Y, Price M, Schmidt DF, Ward M, Nebeker J, Pizer S (2022). Integrated health record viewers and reduction in duplicate medical imaging: retrospective observational analysis. JMIR Med Inform.

[6] Liu Y, Jin H, Yu Z, Tong Y (2024). Impact of internet hospital consultations on outpatient visits and expenses: quasi-experimental study. J Med Internet Res.

[7] Wang K, Zou W, Lai Y (2024). Accessibility, cost, and quality of an online regular follow-up visit service at an internet hospital in China: mixed methods study. J Med Internet Res.

[8] Genereaux BW, Dennison DK, Ho K (2018). DICOMweb™: background and application of the web standard for medical imaging. J Digit Imaging.

[9] Pereira H, Romero L, Miguel Faria P (2025). Web-based DICOM viewers: a survey and a performance classification. J Imaging Inform Med.

[10] Norris EC, Halaska C, Sachs PB, Lin CT, Sanfilippo K, Honce JM (2022). Understanding patient experiences, opinions, and actions taken after viewing their own radiology images online: web-based survey. JMIR Form Res.

[11] Wang J, Goldberg JE, Block T (2024). Patterns of access to radiology reports and images through a patient portal. J Imaging Inform Med.

[12] van der Mee FAM, Ottenheijm RPG, Gentry EGS (2025). The impact of different radiology report formats on patient information processing: a systematic review. Eur Radiol.

[13] Chau M (2025). Alone with the diagnosis: a reflective analysis on imaging report access and emotional burden. Radiography (Lond).

[14] HaoDF Online [Website in Simplified Chinese].

[15] Ahmad NA, Mat Ludin AF, Shahar S, Mohd Noah SA, Mohd Tohit N (2022). Willingness, perceived barriers and motivators in adopting mobile applications for health-related interventions among older adults: a scoping review. BMJ Open.

[16] Lemos M, Henriques AR, Lopes DG (2024). Usability and utility of a mobile app to deliver health-related content to an older adult population: pilot noncontrolled quasi-experimental study. JMIR Form Res.

[17] Grossman LV, Masterson Creber RM, Benda NC, Wright D, Vawdrey DK, Ancker JS (2019). Interventions to increase patient portal use in vulnerable populations: a systematic review. J Am Med Inform Assoc.

[18] Turner K, Clary A, Hong YR, Alishahi Tabriz A, Shea CM (2020). Patient portal barriers and group differences: cross-sectional national survey study. J Med Internet Res.

[19] Wolff JL, Dukhanin V, Burgdorf JG, DesRoches CM (2022). Shared access to patient portals for older adults: implications for privacy and digital health equity. JMIR Aging.

[20] Derry-Vick HM, Heathcote LC, Glesby N (2023). Scanxiety among adults with cancer: a scoping review to guide research and interventions. Cancers (Basel).

[21] Ye A, Deng Y, Li X, Shao G (2024). The impact of informatization development on healthcare services in China. Sci Rep.

[22] Son EH, Nahm ES (2023). Adult patients’ experiences of using a patient portal with a focus on perceived benefits and difficulties, and perceptions on privacy and security: qualitative descriptive study. JMIR Hum Factors.

[23] Bailey JE, Pope RA, Elliott EC, Wan JY, Waters TM, Frisse ME (2013). Health information exchange reduces repeated diagnostic imaging for back pain. Ann Emerg Med.

[24] Wang M, Lu X, Du Y (2025). Digital health governance in China by a whole-of-society approach. NPJ Digit Med.

[25] Li C, Xu X, Zhou G (2019). Implementation of national health informatization in China: survey about the status quo. JMIR Med Inform.

[26] Wu G, Gong M, Wu Y, Liu L, Shi B, Zeng Z (2024). Advancing digital health in China: aligning challenges, opportunities, and solutions with the Global Initiative on Digital Health (GIDH). Health Care Sci.

