# Supplementary material for: Use, Utility, and User Experience of Cloud-Based Medical Imaging in Pulmonary Nodule Care in China: Mixed Methods Study
Source: J Med Internet Res. 2026 Mar 30;28:e86745. doi: 10.2196/86745 (PMC13035031; doi:10.2196/86745)
Supplement: Multimedia Appendix 1 [file jmir-v28-e86745-s001.docx]

**A. Physician Interview Guide — Cloud-Based Medical Imaging (CMI)**

1. **Awareness and usage habits**

- Are you familiar with CMI? How do you understand it?
- In outpatient or inpatient practice, how often do you encounter patients who bring CMI materials?
- In what formats do patients most commonly present CMI (Digital Imaging and Communications in Medicine [DICOM] files, QR codes, web links)?
- In routine practice, do you review these images, rely only on printed reports/films, or use both together?

1. **Perceived impact on clinical care**

- In your view, in what ways has CMI had positive effects?
  *(If the open question yields little, the interviewer may probe: tracking nodule changes, saving visit time, reducing duplicate examinations, improving clinic efficiency.)*
- Which design features make CMI more convenient and improve your user experience?
- Under what circumstances do you find CMI of limited practical value or cumbersome?

1. **Suggestions for improvement**

- Among DICOM files, QR codes, and web links, which format do you prefer and why? Which aspects are most important to you?
- From a physician’s perspective, what problems exist in current CMI workflows or platforms?
- Looking ahead, which areas should be prioritized in the development of CMI systems?

**B. Patient Interview Guide — Cloud-Based Medical Imaging (CMI)**

1. **Background information**

- When did you first discover your pulmonary nodule, and how long has it been?
- Have you sought care at multiple hospitals? If yes, were you asked to repeat CT scans?
- Are you familiar with CMI (for example, DICOM files, QR codes, web links)?
- Have you personally used CMI to view your images? Was it only for showing clinicians, or did you also view them yourself?

1. **Specific experiences with CMI**

- How did you obtain CMI?
- Were you able to open and view the images on a mobile phone or computer without difficulty? What challenges did you encounter?
- During clinic visits, did you actively present CMI to clinicians? Were clinicians willing to use it?
- Compared with traditional films/reports, in what ways did CMI help or create inconvenience?

1. **Perceptions and empowerment**

- Has CMI helped you better understand your condition?
- Has CMI facilitated cross-institution visits or expert consultations?
- Did being able to view the images make you feel more reassured, or did it increase anxiety?

1. **Pain points and recommendations**

- What aspects were most troublesome during use (for example, unsupported formats, complicated procedures, required app downloads, QR code expiration)?
- What improvements would you like hospitals or platforms to make regarding CMI?
