# Supplementary material for: Use, Utility, and User Experience of Cloud-Based Medical Imaging in Pulmonary Nodule Care in China: Mixed Methods Study
Source: J Med Internet Res. 2026 Mar 30;28:e86745. doi: 10.2196/86745 (PMC13035031; doi:10.2196/86745)
Supplement: Multimedia Appendix 2 [file jmir-v28-e86745-s002.docx]

| **Supplementary Table 1. Baseline characteristics for 701 patients in PNs after PSM (1:1 n=582)** | | | | |
| --- | --- | --- | --- | --- |
|  | Overall (n=582) | Users  (n=291, 50%) | Non-users  (n=291, 50%) | *P* value |
| **Age (years)** | 50 (40，58) | 49 (40，58) | 50 (40，58) | .775 |
| **Gender** |  |  |  | .799 |
| Female | 355 (61%) | 176 (60.5%) | 179 (61.5%) |  |
| Male | 227 (39%) | 115 (39.5%) | 112 (38.5%) |  |
| **Time (months)** | 12 (3，32) | 14 (3，32) | 12 (2，33) | .109 |
| **Education attainment** |  |  |  | .897 |
| Primary school | 21 (3.6%) | 8 (2.7%) | 13 (4.5%) |  |
| Junior high school | 63 (10.8%) | 38 (13.1%) | 25 (8.6%) |  |
| Senior high school | 85 (14.6%) | 40 (13.7%) | 45 (15.5%) |  |
| Junior college | 131 (22.5%) | 61 (21.0%) | 70 (24.1%) |  |
| Undergraduate | 225 (38.7%) | 115 (39.5%) | 110 (37.8%) |  |
| Graduate | 57 (9.8%) | 29 (10.0%) | 28 (9.6%) |  |
| CMI, could-based medical images; PNs, pulmonary nodules;PSM, propensity score matching | | | | |
