# Supplementary material for: Use, Utility, and User Experience of Cloud-Based Medical Imaging in Pulmonary Nodule Care in China: Mixed Methods Study
Source: J Med Internet Res. 2026 Mar 30;28:e86745. doi: 10.2196/86745 (PMC13035031; doi:10.2196/86745)
Supplement: Multimedia Appendix 3 [file jmir-v28-e86745-s003.docx]

| **Supplementary Table 3. Covariate balance before and after propensity score matching** | | | | | | |
| --- | --- | --- | --- | --- | --- | --- |
| **Variable** | **Unmatched CMI=Yes (n=404)** | **Unmatched CMI=No (n=297)** | **SMD (Unmatched)** | **Matched CMI=Yes (n=291)** | **Matched CMI=No (n=291)** | **SMD (Matched)** |
| **Age (years)** | 46.8 (12.1) | 50.1 (12.7) | 0.262 | 49.2 (12.3) | 49.6 (12.4) | 0.033 |
| **Sex** | 150 (37.1%) | 116 (39.1%) | 0.040 | 115 (39.5%) | 112 (38.5%) | 0.021 |
| **Education** |  |  | 0.181 |  |  | 0.144 |
| Primary school | 8 (2.0%) | 16 (5.4%) | 0.181 | 8 (2.7%) | 13 (4.5%) | 0.092 |
| Junior high school | 41 (10.1%) | 27 (9.1%) | 0.036 | 38 (13.1%) | 25 (8.6%) | 0.144 |
| Senior high school | 43 (10.6%) | 45 (15.2%) | 0.134 | 40 (13.7%) | 45 (15.5%) | 0.049 |
| Junior college | 79 (19.6%) | 71 (23.9%) | 0.106 | 61 (21.0%) | 70 (24.1%) | 0.074 |
| Undergraduate | 181 (44.8%) | 110 (37.0%) | 0.158 | 115 (39.5%) | 110 (37.8%) | 0.035 |
| Graduate | 52 (12.9%) | 28 (9.4%) | 0.109 | 29 (10.0%) | 28 (9.6%) | 0.012 |
| **Time (months)** | 20.5 (21.4) | 21.6 (33.8) | 0.041 | 21.5 (22.9) | 21.8 (33.9) | 0.010 |
| CMI, cloud-based medical imaging; SMD, standardized mean differences | | | | | | |
