# Supplementary material for: Use, Utility, and User Experience of Cloud-Based Medical Imaging in Pulmonary Nodule Care in China: Mixed Methods Study
Source: J Med Internet Res. 2026 Mar 30;28:e86745. doi: 10.2196/86745 (PMC13035031; doi:10.2196/86745)
Supplement: Multimedia Appendix 4 [file jmir-v28-e86745-s004.docx]

| Supplementary Table 2. Joint display of integrated quantitative and qualitative findings | | |
| --- | --- | --- |
| Quantitative finding | **Qualitative themes & illustrative quote** | **Integrated meta-inference** |
| High CMI availability but underuse: 87.2% obtained CMI, yet only 57.6% reported using it (Table 1). | **Theme 2:** “Some QR codes open directly; others require registration and verification—too many steps.”  **Theme 3**: “Links stop working after a year or two, making longitudinal comparison difficult.” | The gap between having CMI and actually using it seems to come from everyday hurdles. Many patients can obtain CMI, but accessing it takes too many steps, links may stop working, and platforms are not consistent across hospitals. |
| Age gradient: older age independently associated with lower odds of CMI use (multivariable OR 0.985, 95% CI 0.972-0.999). | **Theme 2:** older patients struggled with login/verification/account linking and device/network constraints. | Older participants were less likely to use CMI, which fits what we heard in interviews. Logins, identity checks, and switching between systems were common sticking points, so simplifying the process and allowing family support could reduce this barrier. |
| After PSM, users showed different utilization and cost patterns: more internet hospitals (P=.014), more physicians (P=.024), lower treatment and diagnostic costs (78.7% vs 69.1% spending ¥0-2,500; P=.004), lower travel and accommodation costs (85.6% vs 78.6%; P=.026). | **Theme 1:** “I no longer carry a stack of films—showing the images on my phone is enough.”  **Theme 1:** remote consults become more efficient with shared images. | After matching, CMI users reported different care-seeking and cost patterns. This aligns with interview accounts that having images readily available makes it easier to consult multiple clinicians and may reduce unnecessary repeat steps, although the study design does not allow causal conclusions. |
| No difference in anxiety change after PSM (P=.809). | **Theme 4:** “Seeing my images makes me feel more in control.” vs “The more I look, the more anxious I feel.” | On average, anxiety change did not differ, but interviews suggest mixed experiences. For some, viewing images provides reassurance and control; for others it increases worry, especially without clear explanations. This points to the value of clinician communication and built-in interpretation support. |
| Among users, DICOM vs QR/link modality differences: modality-stratified differences in consulting physicians (P=.017) and disease awareness (P=.004). | **Theme 2 & 3:** QR/link workflows are inconsistent and fragile; platform fragmentation drives inefficiency. | Standardized, interactive access appears more supportive of follow-up comparisons and care coordination. In contrast, QR codes or links can be inconsistent and time-limited, which may discourage sustained use. |
